# An approximate solution for a penny-shaped hydraulic fracture that accounts for fracture toughness, fluid viscosity and leak-off

**DOI:** 10.1098/rsos.160737

**Published:** 2016-12-07

**Authors:** E. V. Dontsov

**Affiliations:** Department of Civil and Environmental Engineering, University of Houston, Houston, TX 77204-4003, USA

**Keywords:** penny-shaped hydraulic fracture, multiscale, leak-off, closed form

## Abstract

This paper develops a closed-form approximate solution for a penny-shaped hydraulic fracture whose behaviour is determined by an interplay of three competing physical processes that are associated with fluid viscosity, fracture toughness and fluid leak-off. The primary assumption that permits one to construct the solution is that the fracture behaviour is mainly determined by the three-process multiscale tip asymptotics and the global fluid volume balance. First, the developed approximation is compared with the existing solutions for all limiting regimes of propagation. Then, a solution map, which indicates applicability regions of the limiting solutions, is constructed. It is also shown that the constructed approximation accurately captures the scaling that is associated with the transition from any one limiting solution to another. The developed approximation is tested against a reference numerical solution, showing that accuracy of the fracture width and radius predictions lie within a fraction of a per cent for a wide range of parameters. As a result, the constructed approximation provides a rapid solution for a penny-shaped hydraulic fracture, which can be used for quick fracture design calculations or as a reference solution to evaluate accuracy of various hydraulic fracture simulators.

## 1. Introduction

Hydraulic fractures are the fluid-filled cracks that propagate under the influence of a fluid pressure acting along the crack’s surface. The most common and well-known application of hydraulic fracturing is the stimulation of oil and gas wells to enhance production of hydrocarbons [1]. Other industrial applications include waste remediation process [2], waste disposal [3] and preconditioning in rock mining [4]. Hydraulic fractures also occur in nature in the process of magma ascent through the lithosphere owing to a buoyancy force [5–8,9] or as fluid-filled cracks in glacier beds [10].

Different hydraulic fracture geometries have been considered over the years. Research efforts shifted from the development of simple models, such as the Khristianovich–Zheltov–Geertsma–De Klerk (KGD) model for a plane-strain crack [11], towards more complex models that consider a planar hydraulic fracture [12–14], multiple hydraulic fractures [15–17] or fracture networks [18]. Detailed reviews of various hydraulic fracturing models can be found in [19–21]. In addition, there are techniques in which cracks are not modelled explicitly, such as the phase field [22,23], distinct element method [24,25] and peridynamics [26]. The primary advantage of such methodologies is the ability to tackle complex crack geometries easier than the conventional methods. At the same time, predictions of such approaches should be thoroughly tested against reference solutions to ensure that the new techniques are able to capture all the features of the conventional approaches.

Even for the simplest geometries, hydraulic fractures are known to obey a complex multiscale behaviour, see e.g. a thorough review paper [27]. This multiscale nature arises both in time, where multiple timescales determine fracture evolution, and space, where the solution may experience variations at different length scales in the tip region. As pointed out in [27], the time and length scales are related; that is, a particular timescale in the fracture evolution corresponds to dominance of one length scale in the tip region. Recognizing the significance and multiscale nature of the tip region, many studies were devoted specifically to quantify hydraulic fracture behaviour in the tip region [28–35]. On the other hand, time evolution and regimes of propagation were studied for a plane-strain KGD fracture in [36–40], and for a penny-shaped hydraulic fracture in [40–44]. A recent review paper [27] provides a more detailed summary of the findings and shows the complexity of the behaviour that occurs even for simple fracture geometries.

In view of the multiscale behaviour of hydraulic fractures, the primary goal of this paper is to quantify such a behaviour for the case of a penny-shaped hydraulic fracture, where the latter is driven by a Newtonian fluid and propagates in a permeable medium assuming no fluid lag. Most of the previous studies that addressed the problem of a penny-shaped fracture focused on the limiting regimes of propagation and asymptotic closed-form solutions (or accurate approximations) for the problem [40–43]. One exception is the study [44], in which a numerical solution for the full problem is obtained. The numerical solution is, however, relatively difficult to obtain owing to the temporal and spatial multiscale nature of the solution. In contrast, this study develops a closed-form approximate solution that provides results virtually instantly and accurately captures the complex behaviour of a radial hydraulic fracture at all length scales and timescales. In particular, the developed solution is able to describe all existing limiting solutions and all possible transitions between them, so that it covers all the trajectories in the parametric space for the problem under consideration. This development is made possible by using a closed-form approximation for the tip asymptotic solution obtained in [34], which is used to approximate fracture width profile. Once the fracture geometry is known, the global fluid volume balance is used to determine behaviour of the solution.

The importance of the obtained solution can be summarized as follows. It first shows that the tip region plays a crucial role in hydraulic fracture modelling. It also allows one to obtain a solution rapidly, which can be useful for quick estimations of fracture geometry for any values of fracture toughness, fluid viscosity and leak-off. Owing to a relatively simple implementation of the solution, it can be used as a reference solution to evaluate accuracy of other hydraulic fracturing simulators and, at the same time, as an initial condition to improve stability of the numerical schemes at early times. Finally, the developed approximation leads to construction of the solution map, which indicates validity regions of the limiting solutions and permits one to easily determine whether the solution for given problem parameters corresponds to one of the limiting cases.

This paper is organized as follows. Section 2 describes governing equations for a radial hydraulic fracture with leak-off. Section 3 outlines procedure for obtaining the approximate solution. Comparison of the developed approximation with the existing limiting solutions is presented in §4. Section 5 contains description of the solution structure, where applicability zones of the limiting solutions are indicated. Finally, §6 evaluates accuracy of the approximation by comparing its predictions to the reference numerical solution, which is followed by the summary.

## 2. Governing equations for a penny-shaped hydraulic fracture

This study considers propagation of an axisymmetric (‘radial’ or ‘penny-shaped’ are also used throughout the paper) hydraulic fracture in a permeable rock [27,44]. There are four primary material parameters that appear in the model, which can be introduced in the scaled form, for convenience, as

2.1 $$\mu^{\prime }=12\mu ,\;E^{\prime }=\frac{E}{1-\nu^{2}},\;K^{\prime }=4{\left(\frac{2}{\pi }\right)}^{1/2}K_{Ic},\;C^{\prime }=2C_{L},$$

where *μ* is the fluid viscosity, *E* is the Young modulus, *ν* is the Poisson’s ratio, *K*_I*c*_ is the mode I fracture toughness of the rock and *C*_L_ is the Carter’s leak-off parameter.

Volume balance for an incompressible Newtonian fluid inside the crack can be written as

2.2 $$\frac{\partial w}{\partial t}+\frac{1}{r}\frac{\partial }{\partial r}(rq)+\frac{C^{\prime }}{\sqrt{t-t_{0}(r)}}=Q_{0}\delta (r),\;q=-\frac{w^{3}}{\mu^{\prime }}\frac{\partial p}{\partial r},$$

where *w*(*r*,*t*) denotes the fracture width, *q* is the flux in the radial direction, the term that contains *C*′ accounts for leak-off via Carter’s model, *t*_0_(*r*) is the time instant at which the fracture front was located at point *r*, *p* is the fluid pressure and *Q*_0_ is the fluid injection rate (assumed to be constant in time).

The elasticity equation, which characterizes elastic equilibrium of the rock, relates the fluid pressure inside the crack to the fracture width as [41,44,45]

2.3 $$p(r,t)=-\frac{E^{\prime }}{2\pi R}\int_{0}^{R}M\left(\frac{r}{R},\frac{r^{\prime }}{R}\right)\frac{\partial w(r^{\prime },t)}{\partial r^{\prime }}\;dr^{\prime },$$

where *R* is the fracture radius, and the kernel is

2.4 $$M(\rho ,s)=\left\{ \begin{array}{ll} \frac{1}{\rho }K\left(\frac{s^{2}}{\rho^{2}}\right)+\frac{\rho }{s^{2}-\rho^{2}}E\left(\frac{s^{2}}{\rho^{2}}\right), & \rho >s,\\ \frac{s}{s^{2}-\rho^{2}}E\left(\frac{\rho^{2}}{s^{2}}\right), & \rho <s. \end{array}\right.$$

The functions *K*(⋅) and *E*(⋅) denote the complete elliptic integrals of the first and the second kind, respectively.

Fracture propagation is modelled by a classical result of the linear elastic fracture mechanics (LEFM), in which the fracture opening in the tip region follows the square root solution [46]

2.5 $$w\to \frac{K^{\prime }}{E^{\prime }}{\left(R-r\right)}^{1/2},\;r\to R,$$

which implies that the stress intensity factor is equal to the fracture toughness for a propagating fracture. The propagation condition (2.5) should also be complemented by a zero flux condition at the fracture tip, i.e. *q*(*R*,*t*)=0.

For future reference, it is useful to consider the global fluid balance, which can be obtained by integrating (2.2) with respect to time and space as

2.6 $$\int_{0}^{R}\left(w(r^{\prime },t)+2C^{\prime }\sqrt{t-t_{0}(r^{\prime })}\right)r^{\prime }\;dr^{\prime }=\frac{Q_{0}t}{2\pi },$$

where the relations *q*(*R*,*t*)=0 and *w*(*R*,*t*)=0 were used to obtain the result.

## 3. Approximate solution for a penny-shaped hydraulic fracture

### 3.1. Outline of the methodology

To construct the approximate solution, it is assumed that the fracture evolution is primarily determined by the near-tip behaviour and the global fluid balance (2.6). In this situation, the approximation for the fracture width can be taken in the form

3.1 $$w(r,t)={\left(\frac{R+r}{2R}\right)}^{\lambda }w_{a}(R-r),$$

where *w*_a_ is the tip asymptotic solution that, in addition to distance to the tip *R*−*r*, depends on the material parameters (2.1) and time through *R*(*t*) and $\dot{R\;}\;(t)$, whereas λ is the parameter that is determined later. Here, the tip asymptotic solution, *w*_a_, is the solution of (2.2), (2.3) and (2.5) in the tip region defined as (*R*−*r*)/*R*≪1, which, as shown in [13], is equivalent to the problem of a semi-infinite hydraulic fracture that propagates steadily under plane-strain elastic conditions [32,34]. As a result, because *w*(*R*−*s*,*t*)=*w*_a_(*s*) for *s*≪*R*, the approximation (3.1) precisely solves the governing equations (2.2), (2.3) and (2.5) in the tip region and approximates the solution inside the fracture away from the tip.

In order to construct the approximation, the closed-form approximation for *w*_a_, obtained in [34], is employed. As shown in [34], the three-process tip asymptotic solution, which captures the effects of fracture toughness, fluid viscosity and leak-off, has the maximum error of 0.14% for all regimes of propagation and all possible transitions between them. One of the properties of the tip solution, *w*_a_, is that $w_{a}(s)\propto s^{\bar{\delta }}$, where *s*=*R*−*r* is the distance from a point inside the fracture to the tip and $\bar{\delta }$ is a slowly varying function, which is also part of the solution (i.e. known). This fact allows to reduce (3.1) to

3.2 $$w(r,t)={\left(\frac{R+r}{2R}\right)}^{\lambda }{\left(1-\frac{r}{R}\right)}^{\bar{\delta }}w_{a}(R).$$

Motivated by the results of the limiting asymptotic solutions, for which *R*(*t*)∝*t*^*α*^ and *α* is a number that is equal to either 1/4, 2/5, or 4/9 [44,27], it is further assumed that *R*(*t*)∝*t*^*α*^, where *α* is a slowly varying function. With this approximation, the function *t*_0_(*r*) is determined from the relation *r*/*R*=(*t*_0_/*t*)^*α*^, which together with (3.2) reduces the global fluid balance (2.6) to

3.3 $$w_{a}(R)\int_{0}^{1}{\left(\frac{1+\rho }{2}\right)}^{\lambda }{\left(1-\rho \right)}^{\bar{\delta }}\rho \;d\rho +2C^{\prime }t^{1/2}\int_{0}^{1}\sqrt{1-\rho^{1/\alpha }}\;\rho \;d\rho =\frac{Q_{0}t}{2\pi R^{2}},$$

where *ρ*=*r*/*R* is the scaled spatial coordinate. The integrals in equation (3.3) can be evaluated, in which case (3.3) reduces to

3.4 $$\begin{array}{rl} & w_{a}(R)2^{1+\bar{\delta }}\left[B_{0}\left(\frac{1}{2};\lambda +2,\bar{\delta }+1\right)-B_{0}\left(\frac{1}{2};\lambda +1,\bar{\delta }+2\right)\right]+2C^{\prime }t^{1/2}\alpha B\left(2\alpha ,\frac{3}{2}\right)=\frac{Q_{0}t}{2\pi R^{2}}, \end{array}$$

where *B*(*a*,*b*) denotes the beta function, and

$$B_{0}(x;a,b)\equiv \int_{x}^{1}t^{a-1}{\left(1-t\right)}^{b-1}\;dt=B(a,b)-B(x;a,b),$$

where *B*(*x*;*a*,*b*) is the incomplete beta function.

Procedure for obtaining the approximate solution can be outlined as follows. First, material parameters (2.1), *Q*_0_ and the function *w*_a_(*R*) should be specified. Note that *w*_a_(*R*) also depends on the material parameters (2.1) and $\dot{R\;}\;=\alpha R/t$. Also note that the expression for $\bar{\delta }$ comes from the tip asymptotic solution. Then, by specifying λ and taking an initial guess for *α*=4/9 (corresponds to zero toughness and zero leak-off solution), equation (3.4) can be solved for *R*(*t*) (e.g. by using Newton’s method). Once *R*(*t*) is obtained, the value of *α* is updated according to α=dlog⁡(R)/dlog⁡(t). After that, equation (3.4) is solved again. The iteration procedure is performed until the desired level of convergence is reached, which is typically achieved quickly owing to relatively modest dependence on *α*. Once *R*(*t*) is calculated, the width can be calculated using (3.2). Efficiency, which is defined as the ratio between the current fracture volume and the total amount of injected fluid, can be calculated as

3.5 $$\eta (t)=\frac{2^{2+\bar{\delta }}\pi R^{2}w_{a}(R)}{Q_{0}t}\left[B_{0}\left(\frac{1}{2};\lambda +2,\bar{\delta }+1\right)-B_{0}\left(\frac{1}{2};\lambda +1,\bar{\delta }+2\right)\right].$$

To calculate fluid pressure, one can substitute (3.2) into (2.3) and evaluate the integral, so that

3.6 $$p=\frac{E^{\prime }w_{a}(R)}{R}F(\lambda ,\bar{\delta },\rho ),\;F(\rho ,\lambda ,\bar{\delta })=\frac{1}{2^{1+\lambda }\pi }\int_{0}^{1}\frac{\partial M(\rho ,s)}{\partial s}{\left(1+s\right)}^{\lambda }{\left(1-s\right)}^{\bar{\delta }}\;ds,$$

where the function $F(\rho ,\lambda ,\bar{\delta })$ can be evaluated numerically, and the kernel *M*(*ρ*,*s*) is given in (2.4). The accuracy of such pressure calculation is examined later in the paper.

It should be noted here that λ is taken as a parameter at this point, and its value can be varied to achieve a better approximation. At the same time, in order to capture the elliptic solution for a uniformly pressurized crack, one should set λ=1/2. As is shown later, the values of λ close to 1/2 provide the most accurate results.

### 3.2. Solution in scaled variables

To solve (3.4) numerically, it is useful to introduce scaling, in which the tip asymptotic solution *w*_a_ is expressed. In particular, as shown in [14,17], the solution *w*_a_ is given implicitly by the equation

3.7 $$\hat{s}=g_{\delta }(\hat{K},\hat{C}),\;\hat{K}=\frac{K^{\prime }R^{1/2}}{E^{\prime }w_{a}},\;\hat{C}=\frac{2C^{\prime }t^{1/2}}{\alpha^{1/2}w_{a}},\;\hat{s}=\frac{\mu^{\prime }\alpha R^{3}}{tE^{\prime }w_{a}^{3}},$$

where *g*_*δ*_ is a known function (see appendix A), and $\dot{R\;}\;=\alpha R/t$ is used for velocity of the crack tip. By introducing the following scales

3.8 $$\begin{array}{rl} t^{*} & =\frac{K^{\prime 12}E^{\prime -10}}{\mu^{\prime 2}{\left(2C^{\prime }\right)}^{6}},\;R^{*}=\frac{K^{\prime 10}E^{\prime -8}}{\mu^{\prime 2}{\left(2C^{\prime }\right)}^{4}},\;w_{a}^{*}=\frac{K^{\prime 6}E^{\prime -5}}{\mu^{\prime }{\left(2C^{\prime }\right)}^{2}}\\ \mathrm{and}\;Q_{0}^{*} & =\frac{2\pi K^{\prime 14}E^{\prime -11}}{\mu^{\prime 3}{\left(2C^{\prime }\right)}^{4}},\;p^{*}=\frac{\mu^{\prime }{\left(2C^{\prime }\right)}^{2}}{K^{\prime 4}E^{\prime -4}}, \end{array}\}$$

and the dimensionless quantities

3.9 $$\hat{t}=\frac{t}{t^{*}}=\alpha \frac{{\hat{C}}^{6}{\hat{s}}^{2}}{{\hat{K}}^{12}},\;\hat{R}=\frac{R}{R^{*}}=\frac{{\hat{C}}^{4}{\hat{s}}^{2}}{{\hat{K}}^{10}},\;{\hat{w}}_{a}=\frac{w_{a}}{w_{a}^{*}}=\frac{{\hat{C}}^{2}\hat{s}}{{\hat{K}}^{6}},\;{\hat{Q}}_{0}=\frac{Q_{0}}{Q_{0}^{*}},\;\hat{p}=\frac{p}{p^{*}},$$

the global fluid balance equation (3.4) can be rewritten as

3.10 $$B(\hat{K},\hat{C},\alpha )=\frac{\alpha {\hat{Q}}_{0}{\hat{K}}^{14}}{{\hat{C}}^{4}{\hat{s}}^{3}},$$

where

3.11 $$B(\hat{K},\hat{C},\alpha )\equiv 2^{1+\bar{\delta }}[B_{0}(\frac{1}{2};\lambda +2,\bar{\delta }+1)-B_{0}(\frac{1}{2};\lambda +1,\bar{\delta }+2)]+\hat{C}\alpha^{3/2}B(2\alpha ,\frac{3}{2}),$$

the parameter $\lambda (\hat{K},\hat{C},\alpha )$ is specified later, and $\bar{\delta }=\frac{1}{2}(1+\Delta (\hat{K},\hat{C}))$ (see appendix A). The first equation in (3.7), the first relation in (3.9) and (3.10) can be combined to yield the system of equations

3.12 $$\begin{array}{rl} & \alpha^{1/2}{\hat{C}}^{3}g_{\delta }(\hat{K},\hat{C})-{\hat{t}}^{1/2}{\hat{K}}^{6}=0\\ \mathrm{and}\; & {\hat{t}}^{3/2}{\hat{K}}^{4}B(\hat{K},\hat{C},\alpha )-\alpha^{5/2}{\hat{Q}}_{0}{\hat{C}}^{5}=0. \end{array}\}$$

To obtain the solution, the system of equations (3.12) is solved numerically for $\hat{K}(\hat{t})$ and $\hat{C}(\hat{t})$ using Newton’s method. Note that ${\hat{Q}}_{0}$ is a parameter, and the initial value of *α* is taken as 4/9, which is then updated iteratively using $\alpha =d\log(\hat{R})/d\log(\hat{t})$ (so that the system (3.12) is solved a few times until *α* converges).

Once the time histories $\hat{K}(\hat{t})$ and $\hat{C}(\hat{t})$ are obtained, equation (3.7) is used to find $\hat{s}(\hat{t})$, and the relations in (3.9) are used to calculate $\hat{R}(\hat{t})$ and ${\hat{w}}_{a}(\hat{t})$. The values of $\bar{\delta }$ and λ (see §4.5) are also expressed in terms of $\hat{K}(\hat{t})$ and $\hat{C}(\hat{t})$. The pressure and the fracture opening solutions can be obtained from (3.6) and (3.2), respectively, as

3.13 $$\hat{p}(\rho ,\hat{t})=\frac{{\hat{w}}_{a}(\hat{t})}{\hat{R}(t)}F(\rho ,\lambda ,\bar{\delta }),\;\hat{w}(\rho ,\hat{t})=2^{-\lambda }{\left(1-\rho \right)}^{\bar{\delta }}{\left(1+\rho \right)}^{\lambda }{\hat{w}}_{a}(\hat{t}),\;\rho =\frac{r}{R}=\frac{\hat{r}}{\hat{R}}\;,$$

whereas the efficiency can be expressed using (3.5), (3.10) and (3.11) as

3.14 $$\eta (\hat{t})=1-\frac{\hat{C}\alpha^{3/2}B(2\alpha ,\frac{3}{2})}{B(\hat{K},\hat{C},\alpha )}.$$

Finally, the solution is given in terms of the fracture radius $\hat{R}(\hat{t})$, fracture width $\hat{w}(\rho ,\hat{t})$, fluid pressure $\hat{p}(\rho ,\hat{t})$ and efficiency $\eta (\hat{t})$. Equations (3.8) and (3.9) can be used to convert the solution to the unscaled form if needed.

## 4. Comparison with vertex solutions

The problem of a penny-shaped fracture with fluid loss and no lag has four limiting regimes of propagation (or vertex solutions) [44,27]: the storage viscosity (*M*), storage toughness (*K*), leak-off viscosity ($\tilde{M}$) and leak-off toughness ($\tilde{K}$). These limiting regimes are determined by dominance of one of the dissipative mechanisms associated with fluid viscosity and rock toughness, and by dominance of either the fluid storage in the fracture or fluid leak-off in the surrounding rock. The limiting regimes can be expressed using the two variables $0\leq \hat{K}\leq 1$ and 0≤*η*≤1 as

4.1 $$M:\;\hat{K}=0,\;\eta =1,\;\tilde{M}:\;\hat{K}=0,\;\eta =0,\;K:\;\hat{K}=1,\;\eta =1,\;\tilde{K}:\;\hat{K}=1,\;\eta =0.$$

Indeed, the case $\hat{K}=0$ corresponds to the situation of no fracture toughness, while $\hat{K}=1$ implies that the tip asymptotic solution follows the LEFM square root solution and hence, the effect of fluid viscosity is negligible. At the same time, the efficiency *η*=1 corresponds to the situation in which all fluid is stored in the fracture, whereas *η*=0 signifies the case in which all the injected fluid leaks into the formation. It is important to note that *η*=1 corresponds to $\hat{C}=0$ and *η*=0 corresponds to $\hat{C}\to \infty$, as can be seen from (3.11) and (3.14).

To evaluate accuracy of the approximate solution, and to specify the values for λ, the remainder of this section is devoted to a comparison between the limiting regimes (4.1) of the approximate solution and the existing reference solutions for these cases. In addition, setting some of the parameters, such as *K*′ and *C*′ (or $\hat{K}$ and $\hat{C}$), to zero makes the scaling (3.8) and (3.9) deficient. It is still possible, however, to use a combination of variables, such as $\hat{R}/{\hat{w}}_{a}^{2}$, because it may not depend on $\hat{C}$ and, consequently, on *C*′. This fact will be used for the remaining part of this section to write the limiting solutions in terms of scaling (3.8) and (3.9).

### 4.1. *M* vertex limit of the solution

Before proceeding with the *M* vertex limit, it is useful to rewrite equations (3.9) and (3.12) in the form

4.2 $${\hat{w}}_{a}={\left(\frac{\alpha^{2}}{g_{\delta }{\left(\hat{K},\hat{C}\right)}^{2}B{\left(\hat{K},\hat{C},\alpha \right)}^{3}}\right)}^{1/9}{\hat{Q}}_{0}^{1/3}{\hat{t}}^{1/9},\;\hat{R}={\left(\frac{g_{\delta }(\hat{K},\hat{C})}{\alpha B{\left(\hat{K},\hat{C},\alpha \right)}^{3}}\right)}^{1/9}{\hat{Q}}_{0}^{1/3}{\hat{t}}^{4/9}.$$

Because the *M* vertex solution corresponds to the case of no toughness and no leak-off, it is possible to set $\hat{K}=\hat{C}=0$ in (4.2), in which case *α*=4/9. Note that B(0,0,4/9)=0.1979 and $g_{\delta }(0,0)=1/\beta_{m}^{3}=0.0321$ (see appendix A) are constants, where *β*_*m*_=2^1/3^3^5/6^.

By introducing scaling that is associated with the *M* vertex solution [13],^41^,^44^]

4.3 $$\gamma_{m}=\frac{R}{L_{m}},\;\Omega_{m}=\frac{w}{\varepsilon_{m}L_{m}},\;\Pi_{m}=\frac{p}{\varepsilon_{m}E^{\prime }},\;\varepsilon_{m}={\left(\frac{\mu^{\prime }}{E^{\prime }t}\right)}^{1/3},\;L_{m}={\left(\frac{Q_{0}^{3}E^{\prime }t^{4}}{\mu^{\prime }}\right)}^{1/9},$$

equations (4.2) can be reduced to

4.4 $$\Omega_{a,m}={\left(\frac{2}{3^{4}\pi^{3}g_{\delta }{\left(0,0\right)}^{2}B{\left(0,0,4/9\right)}^{3}}\right)}^{1/9},\;\gamma_{m}={\left(\frac{3^{2}g_{\delta }(0,0)}{2^{5}\pi^{3}B{\left(0,0,4/9\right)}^{3}}\right)}^{1/9},$$

where *α*=4/9 (because *L*_*m*_∝*t*^4/9^) is used to obtain the result. The solutions for the fracture width and pressure variations can be written using (3.13) as

4.5 $$\Omega_{m}(\rho )=\Omega_{a,m}2^{-\lambda_{m}}{\left(1+\rho \right)}^{\lambda_{m}}{\left(1-\rho \right)}^{2/3},\;\Pi_{m}(\rho )=\frac{\Omega_{a,m}}{\gamma_{m}}F\left(\rho ,\lambda_{m},\frac{2}{3}\right),$$

where the fact that $\bar{\delta }=\frac{1}{2}(1+\Delta (0,0))=\frac{2}{3}$ is used. Comparison of the fracture width at the wellbore *Ω*_*m*_(0) and fracture radius *γ*_*m*_ that are calculated using (4.4) and (4.5) with those obtained in [41] for the *M* vertex case implies that λ_*m*_=0.487 provides the best approximation, for which the error for both *Ω*_*m*_(0) and *γ*_*m*_ is below 0.5%.

The *M* vertex limit of the approximate solution can be summarized as

4.6 $$\Omega_{m}(\rho )=1.1901{\left(1+\rho \right)}^{0.487}{\left(1-\rho \right)}^{2/3},\;\Pi_{m}(\rho )=2.4019F(\rho ,0.487,\frac{2}{3}),\;\gamma_{m}=0.6944,$$

which can be written in the dimensional form using (4.3) as

4.7 $$\begin{array}{rl} w_{m}(\rho ,t) & =1.1901{\left(\frac{\mu^{\prime 2}Q_{0}^{3}t}{E^{\prime 2}}\right)}^{1/9}{\left(1+\rho \right)}^{0.487}{\left(1-\rho \right)}^{2/3},\\ p_{m}(\rho ,t) & =2.4019{\left(\frac{\mu^{\prime }E^{\prime 2}}{t}\right)}^{1/3}F\left(\rho ,0.487,\frac{2}{3}\right)\\ \mathrm{and}\;R_{m}(t) & =0.6944{\left(\frac{Q_{0}^{3}E^{\prime }t^{4}}{\mu^{\prime }}\right)}^{1/9}, \end{array}\}$$

where the function F is defined in (3.6).

### 4.2. $\tilde{M}$ vertex limit of the solution

In order to obtain the $\tilde{M}$ limit, one should consider the limiting case of no fracture toughness, i.e. $\hat{K}=0$, and zero efficiency, i.e. *η*=0. In this situation, equation (3.14) implies that

4.8 $$B(0,\hat{C},\alpha )=\hat{C}\alpha^{3/2}B(2\alpha ,\frac{3}{2}).$$

Because the zero efficiency case corresponds to large values of $\hat{C}$, one can use the asymptotic behaviour of *g*_*δ*_ at large $\hat{C}$, namely $\hat{s}=g_{\delta }(0,\hat{C})=1/(\beta_{\tilde{m}}^{4}\hat{C})$ (see appendix A), where $\beta_{\tilde{m}}=4/15^{1/4}/(\sqrt{2}-1{)}^{1/4}$. As a result, equations (3.9) and (3.12) can be rewritten in the form

4.9 $$\hat{t}=\frac{\alpha \beta_{\tilde{m}}^{8}{\hat{R}}_{\tilde{m}}^{6}}{{\hat{w}}_{a,\tilde{m}}^{8}},\;\hat{C}=\frac{\beta_{\tilde{m}}^{4}{\hat{R}}_{\tilde{m}}^{3}}{{\hat{w}}_{a,\tilde{m}}^{5}},\;B(0,\hat{C},\alpha )=\frac{\alpha {\hat{Q}}_{0}\beta_{\tilde{m}}^{8}{\hat{R}}_{\tilde{m}}^{4}}{{\hat{w}}_{a,\tilde{m}}^{9}}.$$

The combination of (4.8) and (4.9) finally leads to

4.10 $${\hat{R}}_{\tilde{m}}={\left(\frac{{\hat{Q}}_{0}}{\alpha B(2\alpha ,\frac{3}{2})}\right)}^{1/2}{\hat{t}}^{1/4},\;{\hat{w}}_{a,\tilde{m}}=\frac{\beta_{\tilde{m}}}{\alpha^{1/4}}{\left(\frac{{\hat{Q}}_{0}}{B(2\alpha ,\frac{3}{2})}\right)}^{3/8}{\hat{t}}^{1/16},$$

in which case it is clear that $\alpha =d\log({\hat{R}}_{\tilde{m}})/d\log(\hat{t})=1/4$, and hence the beta function can be evaluated as $B(\frac{1}{2},\frac{3}{2})=\pi /2$.

By introducing scaling that is associated with the $\tilde{M}$ vertex solution [44,13]

4.11 $$\gamma_{\tilde{m}}=\frac{R}{L_{\tilde{m}}},\;\Omega_{\tilde{m}}=\frac{w}{\varepsilon_{\tilde{m}}L_{\tilde{m}}},\;\Pi_{\tilde{m}}=\frac{p}{\varepsilon_{\tilde{m}}E^{\prime }},\;\varepsilon_{\tilde{m}}={\left(\frac{\mu^{\prime 4}C^{\prime 6}}{E^{\prime 4}Q_{0}^{2}t^{3}}\right)}^{1/16},\;L_{\tilde{m}}={\left(\frac{Q_{0}^{2}t}{C^{\prime 2}}\right)}^{1/4},$$

relations (4.10) can be reduced to

4.12 $$\Omega_{a,\tilde{m}}=\frac{\beta_{\tilde{m}}2^{3/8}}{\pi^{3/4}},\;\gamma_{\tilde{m}}=\frac{\sqrt{2}}{\pi },$$

and solutions for the fracture width and pressure variations can be written using (3.13) as

4.13 $$\Omega_{\tilde{m}}(\rho )=\Omega_{a,\tilde{m}}2^{-\lambda_{\tilde{m}}}{\left(1+\rho \right)}^{\lambda_{\tilde{m}}}{\left(1-\rho \right)}^{5/8},\;\Pi_{\tilde{m}}(\rho )=\frac{\Omega_{a,\tilde{m}}}{\gamma_{\tilde{m}}}F\left(\rho ,\lambda_{\tilde{m}},\frac{5}{8}\right),$$

where $\bar{\delta }=5/8$ is used, which corresponds to the case $\hat{K}=0$ and $\hat{C}\to \infty$. Comparison of the fracture width at the wellbore $\Omega_{\tilde{m}}(0)$ and the fracture radius $\gamma_{\tilde{m}}$ in (4.13) with the reference solution for the $\tilde{M}$ vertex [44,13] indicates that $\lambda_{\tilde{m}}=0.397$ provides the most accurate approximation, in which the error of $\Omega_{\tilde{m}}(0)$ is below 0.1%, whereas the value of $\gamma_{\tilde{m}}$ is captured precisely.

The $\tilde{M}$ vertex limit of the approximate solution can be summarized from (4.12) and (4.13) as

4.14 $$\Omega_{\tilde{m}}(\rho )=1.0574{\left(1+\rho \right)}^{0.397}{\left(1-\rho \right)}^{5/8},\;\Pi_{\tilde{m}}(\rho )=3.0931F(\rho ,0.397,\frac{5}{8}),\;\gamma_{\tilde{m}}=0.4502,$$

and can be written in the dimensional form using (4.11) as

4.15 $$\begin{array}{rl} w_{\tilde{m}}(\rho ,t) & =1.0574{\left(\frac{\mu^{\prime 4}Q_{0}^{6}t}{E^{\prime 4}C^{\prime 2}}\right)}^{1/16}{\left(1+\rho \right)}^{0.397}{\left(1-\rho \right)}^{5/8},\\ p_{\tilde{m}}(\rho ,t) & =3.0931{\left(\frac{\mu^{\prime 4}E^{\prime 12}C^{\prime 6}}{Q_{0}^{2}t^{3}}\right)}^{1/16}F\left(\rho ,0.397,\frac{5}{8}\right)\\ \mathrm{and}\;R_{\tilde{m}}(t) & =0.4502{\left(\frac{Q_{0}^{2}t}{C^{\prime 2}}\right)}^{1/4}, \end{array}\}$$

where the function F is defined in (3.6).

### 4.3. *K* vertex limit of the solution

The *K* vertex solution corresponds to the case when $\hat{K}=1$ and *η*=1 (or $\hat{C}=0$). In this situation, $\hat{s}=g_{\delta }(1,0)=0$ and equations (3.9) and (3.12) can be rewritten in the form

4.16 $${\hat{w}}_{a,k}={\left(\frac{{\hat{Q}}_{0}\hat{t}}{B(1,0,\alpha )}\right)}^{1/5},\;{\hat{R}}_{k}={\left(\frac{{\hat{Q}}_{0}\hat{t}}{B(1,0,\alpha )}\right)}^{2/5},$$

which implies that *α*=2/5.

By introducing scaling that is associated with the *K* vertex solution [13,41,44]

4.17 $$\gamma_{k}=\frac{R}{L_{k}},\;\Omega_{k}=\frac{w}{\varepsilon_{k}L_{k}},\;\Pi_{k}=\frac{p}{\varepsilon_{k}E^{\prime }},\;\varepsilon_{k}={\left(\frac{K^{\prime 6}}{E^{\prime 6}Q_{0}t}\right)}^{1/5},\;L_{k}={\left(\frac{Q_{0}E^{\prime }t}{K^{\prime }}\right)}^{2/5},$$

relations (4.16) reduce to

4.18 $$\Omega_{a,k}={\left(\frac{3}{\sqrt{2}\pi }\right)}^{1/5},\;\gamma_{k}={\left(\frac{3}{\sqrt{2}\pi }\right)}^{2/5},$$

in which case the width and pressure spatial variations can be obtained from (3.13) as

4.19 $$\Omega_{k}(\rho )=\Omega_{a,k}2^{-\lambda_{k}}{\left(1+\rho \right)}^{\lambda_{k}}{\left(1-\rho \right)}^{1/2},\;\Pi_{k}(\rho )=\frac{\Omega_{a,k}}{\gamma_{k}}F\left(\rho ,\lambda_{k},\frac{1}{2}\right),$$

where $\bar{\delta }=\frac{1}{2}(1+\Delta (1,0))=\frac{1}{2}$ was used. Because the fracture width for the *K* vertex solution is an ellipse [41], then λ_*k*_=1/2 leads to the exact solution.

Given that $F(\rho ,\frac{1}{2},\frac{1}{2})=\pi /(8\sqrt{2})$ and $B(1,0,\alpha )=(3\sqrt{2}{)}^{-1}$, the solution can be summarized as

4.20 $$\Omega_{k}(\rho )={\left(\frac{3}{8\pi }\right)}^{1/5}{\left(1-\rho^{2}\right)}^{1/2},\;\Pi_{k}(\rho )=\frac{\pi }{8}{\left(\frac{\pi }{12}\right)}^{1/5},\;\gamma_{k}={\left(\frac{3}{\sqrt{2}\pi }\right)}^{2/5},$$

which coincides with the *K* vertex solution in [41,44]. The above results can be written in the unscaled form as

4.21 $$\begin{array}{rl} w_{k}(\rho ,t) & =0.6537{\left(\frac{K^{\prime 4}Q_{0}t}{E^{\prime 4}}\right)}^{1/5}{\left(1-\rho^{2}\right)}^{1/2},\\ p_{k}(\rho ,t) & =0.3004{\left(\frac{K^{\prime 6}}{E^{\prime }Q_{0}t}\right)}^{1/5}\\ \mathrm{and}\;R_{k}(t) & =0.8546{\left(\frac{E^{\prime }Q_{0}t}{K^{\prime }}\right)}^{2/5}. \end{array}\}$$

Note that because the fracture width for the *K* vertex limit is an ellipse and that the approximation (3.1) captures elliptic width profile precisely, the obtained limited solution is the exact solution for the *K* vertex limit and should not be tested against a reference solution.

### 4.4. $\tilde{K}$ vertex limit of the solution

The $\tilde{K}$ vertex limit of the solution corresponds to the situation for which $\hat{K}=1$ and *η*=0. As a result, the efficiency relation (3.14) reduces to

4.22 $$B(1,\hat{C})=\hat{C}\alpha^{3/2}B(2\alpha ,\frac{3}{2}),$$

and equations (3.9) and (3.12) can be rewritten as

4.23 $${\hat{w}}_{a,\tilde{k}}={\left(\frac{{\hat{Q}}_{0}{\hat{t}}^{1/2}}{\alpha B(2\alpha ,\frac{3}{2})}\right)}^{1/4},\;{\hat{R}}_{\tilde{k}}={\left(\frac{{\hat{Q}}_{0}{\hat{t}}^{1/2}}{\alpha B(2\alpha ,\frac{3}{2})}\right)}^{1/2}.$$

As for the $\tilde{M}$ solution, $\alpha =d\log({\hat{R}}_{\tilde{k}})/d\log(\hat{t})=1/4$, and the beta function can be evaluated as $B(\frac{1}{2},\frac{3}{2})=\pi /2$.

By introducing scaling that is associated with the $\tilde{K}$ vertex solution [13,42,44]

4.24 $$\gamma_{\tilde{k}}=\frac{R}{L_{\tilde{k}}},\;\Omega_{\tilde{k}}=\frac{w}{\varepsilon_{\tilde{k}}L_{\tilde{k}}},\;\Pi_{\tilde{k}}=\frac{p}{\varepsilon_{\tilde{k}}E^{\prime }},\;\varepsilon_{\tilde{k}}={\left(\frac{K^{\prime 8}C^{\prime 2}}{E^{\prime 8}Q_{0}^{2}t}\right)}^{1/8},\;L_{\tilde{k}}={\left(\frac{Q_{0}^{2}t}{C^{\prime 2}}\right)}^{1/4},$$

relations (4.23) can be reduced to

4.25 $$\Omega_{a,\tilde{k}}={\left(\frac{\sqrt{2}}{\pi }\right)}^{1/2},\;\gamma_{\tilde{k}}=\frac{\sqrt{2}}{\pi },$$

and the width and pressure spatial variations can be obtained using (3.13) as

4.26 $$\Omega_{\tilde{k}}(\rho )=\Omega_{a,\tilde{k}}2^{-\lambda_{\tilde{k}}}{\left(1+\rho \right)}^{\lambda_{\tilde{k}}}{\left(1-\rho \right)}^{1/2},\;\Pi_{\tilde{k}}(\rho )=\frac{\Omega_{a,\tilde{k}}}{\gamma_{\tilde{k}}}F(\rho ,\lambda_{\tilde{k}},\frac{1}{2}),$$

where, similarly to the *K* vertex limit, $\bar{\delta }=\frac{1}{2}$ was used. As for the *K* vertex case, the fracture width for the $\tilde{K}$ vertex solution is an ellipse [42,44], and $\lambda_{\tilde{k}}=1/2$ leads to the exact solution.

Because $F(\rho ,\frac{1}{2},\frac{1}{2})=\pi /(8\sqrt{2}),$ the results in (4.23) and (4.26) can be summarized as

4.27 $$\Omega_{\tilde{k}}(\rho )=\frac{{\left(1-\rho^{2}\right)}^{1/2}}{2^{1/4}\pi^{1/2}},\;\Pi_{\tilde{k}}(\rho )=\frac{\pi^{3/2}}{2^{15/4}},\;\gamma_{\tilde{k}}=\frac{2^{1/2}}{\pi },$$

which coincides with the $\tilde{K}$ vertex solution in [42,44]. The unscaled form of (4.27) is

4.28 $$\begin{array}{rl} w_{\tilde{k}}(\rho ,t) & =0.4744{\left(\frac{K^{\prime 8}Q_{0}^{2}t}{E^{\prime 8}C^{\prime 2}}\right)}^{1/8}{\left(1-\rho^{2}\right)}^{1/2},\\ p_{\tilde{k}}(\rho ,t) & =0.4139{\left(\frac{K^{\prime 8}C^{\prime 2}}{Q_{0}^{2}t}\right)}^{1/8}\\ \mathrm{and}\;R_{\tilde{k}}(t) & =0.4502{\left(\frac{Q_{0}^{2}t}{C^{\prime 2}}\right)}^{1/4}. \end{array}\}$$

Similar to the *K* vertex solution, the $\tilde{K}$ limit of the approximate solution coincides with the $\tilde{K}$ limit of the exact solution, because the approximation (3.1) captures elliptic width profile precisely.

### 4.5. Parameter λ interpolation

This section aims to describe an interpolation scheme to determine values of the parameter λ, knowing its values for the limiting cases. Comparison between the developed approximate solution and the vertex solutions indicates that λ_*m*_=0.487, $\lambda_{\tilde{m}}=0.397$, λ_*k*_=0.5 and $\lambda_{\tilde{k}}=0.5$. Owing to a small variation of λ, one may consider the following interpolation procedure:

4.29 $$\lambda (\hat{K},\hat{C},\alpha )=\lambda_{m}(1-{\hat{K}}^{4})\eta_{0}+\lambda_{k}{\hat{K}}^{4}\eta_{0}+\lambda_{\tilde{m}}(1-{\hat{K}}^{4})(1-\eta_{0})+\lambda_{\tilde{k}}{\hat{K}}^{4}(1-\eta_{0}),$$

where $\eta_{0}(\hat{K},\hat{C},\alpha )$ is the approximation for the efficiency (3.14) for which λ=0.5 is used. The interpolation (4.29) captures the correct values of λ at all four vertices, and provides an approximation elsewhere. It is important to note that the choice of ${\hat{K}}^{4}$ in lieu of e.g. $\hat{K}$ is due to the fact that the function *g*_*δ*_ depends on ${\hat{K}}^{4}$ when $\hat{C}\to \infty$ (transition between $\tilde{M}$ and $\tilde{K}$ regimes); see appendix A. Note that the transition from *M* to *K* (*η*=0) is less important for the interpolation purposes, because λ_*k*_≈λ_*m*_ and any interpolation would work. The transitions from *M* to $\tilde{M}$ and from *K* to $\tilde{K}$ are determined by the efficiency, hence the efficiency is used as a parameter for the interpolation in (4.29). Because even a constant approximation λ≡0.5 is able to provide relatively accurate approximation owing to a small variation of λ, the accuracy of the interpolation (4.29) is expected to be sufficient. This statement will be verified later in §6, where the approximate solution is compared with the reference numerical solution.

## 5. Structure of the solution

Solution in the original scaling is given by the quantities $\hat{R}$, $\hat{w}$, $\hat{p}$ and *η*, which depend on the dimensionless time $\hat{t}$ and the parameter $\hat{Q}$. At this point, it is useful to rewrite all the variables in terms of the scaling that will be used in the reference numerical solution in §6. In particular, the scaling of the numerical solution is determined by the following quantities [44]

5.1 $$\begin{array}{rl} \varepsilon & ={\left(\frac{\mu^{\prime }}{E^{\prime }t_{mk}}\right)}^{1/3}=\frac{w_{a}^{*}}{2^{2}\phi^{1/2}R^{*}},\;L={\left(\frac{Q_{0}^{3}E^{\prime }t_{mk}^{4}}{\mu^{\prime }}\right)}^{1/9}=2^{4}\phi R^{*}\\ \mathrm{and}\;t_{mk} & ={\left(\frac{\mu^{\prime 5}E^{\prime 13}Q_{0}^{3}}{K^{\prime 18}}\right)}^{1/2}=2^{6}\phi^{3/2}t^{*}, \end{array}\}$$

whereas the leak-off parameter *ϕ* is defined as

5.2 $$\phi =\frac{\mu^{\prime 3}E^{\prime 11}C^{\prime 4}Q_{0}}{K^{\prime 14}}=\frac{\pi }{8}{\hat{Q}}_{0}.$$

In this setting, the relationship between the new scaling and the original scaling (3.8) and (3.9) can be summarized as

5.3 $$\tau =\frac{t}{t_{mk}}=\frac{\hat{t}}{2^{6}\phi^{3/2}},\;\gamma =\frac{R}{L}=\frac{\hat{R}}{2^{4}\phi },\;\Omega =\frac{w}{\varepsilon L}=\frac{\hat{w}}{2^{2}\phi^{1/2}},\;\Pi =\frac{p}{\varepsilon E^{\prime }}=2^{2}\phi^{1/2}\hat{p},$$

where the solution in new scaling is given by the fracture radius *γ*(*τ*), fracture width *Ω*(*ρ*,*τ*), fluid pressure *Π*(*ρ*,*τ*) and efficiency *η*(*τ*).

**Figure 1.**
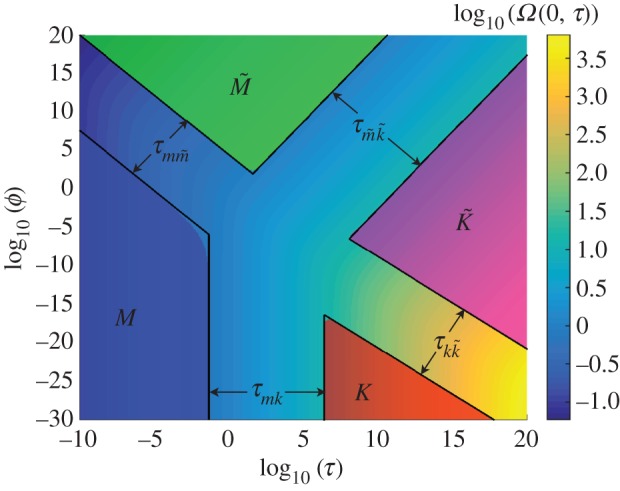
Variation of the approximate fracture width solution *Ω*(0,*τ*) versus dimensionless time *τ* and leak-off parameter *ϕ*. Blue, red, green and magenta regions indicate, respectively, validity zones of the *M* vertex solution (4.6)–(4.7), *K* vertex solution (4.20)–(4.21), $\tilde{M}$ vertex solution (4.14)–(4.15) and $\tilde{K}$ vertex solution (4.27)–(4.28), according to (5.4). Black lines indicate boundaries of applicability of the vertex solutions.

To visualize the structure of the solution, figure 1 plots the approximate width solution evaluated at the wellbore *Ω*(0,*τ*) versus *τ* for a range of values *ϕ*. Blue, red, green and magenta regions indicate, respectively, validity zones of the *M* vertex solution (4.6)–(4.7), *K* vertex solution (4.20)–(4.21), $\tilde{M}$ vertex solution (4.14)–(4.15) and $\tilde{K}$ vertex solution (4.27)–(4.28). These validity zones are defined as

5.4 $$\sqrt{{\left(\frac{\Omega (0,\tau )-\Omega_{i}(0,\tau )}{\Omega (0,\tau )}\right)}^{2}+{\left(\frac{\gamma (\tau )-\gamma_{i}(\tau )}{\gamma (\tau )}\right)}^{2}}<10^{-2},$$

where *i* corresponds to either *M*, *K*, $\tilde{M}$ or $\tilde{K}$ vertex solutions. Note that all quantities in formula (5.4) are written in terms of the scaling (5.3).

Figure 1 is a map of the solutions and allows one to determine the regime of propagation of a radial fracture for a given problem parameters. For instance, for the parameters *E*′=10 GPa, *K*′=1 MPa⋅m^1/2^, *Q*_0_=10^−2^ m^3^ s^−1^, *t*=10^3^ s, *μ*′=1 Pa⋅s and *C*′=10^−6^ m s^−1/2^, the dimensionless time and leak-off parameters, respectively, are *τ*=10^−5^ and *ϕ*=1, in which case, according to the figure 1, the *M* vertex solution still can be used to estimate the fracture parameters. At the same time, if the viscosity is changed to *μ*′=0.01 Pa⋅s (other parameters are kept the same), then *τ*=1 and *ϕ*=10^−6^, so that none of the vertex solutions can be used. Instead, the developed approximation can be used. In addition, figure 1 can be used to indicate possible time evolution paths. The fracture starts at the *M* vertex and ultimately reaches the $\tilde{K}$ vertex. Along its way, the fracture may pass through the *K* vertex, through the $\tilde{M}$ vertex, or be in the transition region between the two, as was described in [44,27]. Black lines in figure 1 show the boundaries of applicability of the vertex solutions and are determined next. Note that all the timescales that are associated with the transitions from one vertex solution to another (obtained in the remainder of this section using limits of the approximate solution) are consistent with the results in [44], which were obtained using a different method. This indicates that the developed approximate solution precisely captures the scaling that is associated with the transitions from one vertex solution to another.

*Transition along the MK edge.* To quantify the boundaries between the *M* and *K* vertices, it is necessary to consider the *MK* edge transition. This *MK* edge solution corresponds to the situation of no leak-off, or *η*=1. As a result, $\hat{C}=0$ and equations (3.12) and (3.14) reduce to

5.5 $$\frac{{\hat{t}}^{2}}{{\hat{Q}}_{0}^{3}}=\frac{\alpha^{5}{\hat{K}}^{18}}{B{\left(\hat{K},0,\alpha \right)}^{3}g_{\delta }{\left(\hat{K},0\right)}^{5}}.$$

Solution of equation (5.5) determines the *MK* edge solution. It is important to note that the solution depends on the parameter $\hat{t}/{\hat{Q}}^{3/2}$, which can be rewritten using (5.2) and (5.3) as *τ*. As a result, the dimensionless time that quantifies the *MK* transition is

5.6 $$\tau_{mk}=\frac{t}{t_{mk}}=\tau ,\;t_{mk}={\left(\frac{\mu^{\prime 5}E^{\prime 13}Q_{0}^{3}}{K^{\prime 18}}\right)}^{1/2}.$$

The associated boundaries of the *MK* transition, shown in figure 1, are calculated numerically as

5.7 $$\tau_{mk}^{\left(1\right)}\approx 4.54\times 10^{-2},\;\tau_{mk}^{\left(2\right)}\approx 2.59\times 10^{6},$$

where $\tau_{mk}^{\left(1\right)}$ corresponds to the beginning of the *MK* transition (near the *M* vertex), whereas $\tau_{mk}^{\left(2\right)}$ corresponds to the end of the transition (near the *K* vertex).

*Transition along the* $M\tilde{M}$ *edge.* To quantify the boundaries between the *M* and $\tilde{M}$ vertices, it is necessary to consider the $M\tilde{M}$ edge transition. This $M\tilde{M}$ edge solution corresponds to the situation in which $\hat{K}=0$. As a result, equations (3.12) reduce to

5.8 $$\frac{{\hat{t}}^{7}}{{\hat{Q}}_{0}^{6}}=\frac{\alpha^{13}{\hat{C}}^{18}}{g_{\delta }{\left(0,\hat{C}\right)}^{4}B{\left(0,\hat{C},\alpha \right)}^{6}},$$

which determines the $M\tilde{M}$ edge solution. The solution depends on the parameter $\hat{t}/{\hat{Q}}^{6/7}$, which can be rewritten using (5.2) and (5.3) as *τϕ*^9/14^. In this case, the dimensionless time that quantifies the $M\tilde{M}$ transition is

5.9 $$\tau_{m\tilde{m}}=\frac{t}{t_{m\tilde{m}}}=\tau \phi^{9/14},\;t_{m\tilde{m}}={\left(\frac{\mu^{\prime 2}Q_{0}^{3}}{E^{\prime 2}C^{\prime 9}}\right)}^{2/7}.$$

The associated boundaries of the $M\tilde{M}$ transition, shown in figure 1, are calculated numerically as

5.10 $$\tau_{m\tilde{m}}^{\left(1\right)}\approx 7.41\times 10^{-6},\;\tau_{m\tilde{m}}^{\left(2\right)}\approx 7.20\times 10^{2},$$

where $\tau_{m\tilde{m}}^{\left(1\right)}$ corresponds to the beginning of the $M\tilde{M}$ transition (near the *M* vertex), whereas $\tau_{m\tilde{m}}^{\left(2\right)}$ corresponds to the end of the transition (near the $\tilde{M}$ vertex).

*Transition along the* $K\tilde{K}$ *edge.* To quantify the boundaries between the *K* and $\tilde{K}$ vertices, it is necessary to consider the $K\tilde{K}$ edge transition. This $K\tilde{K}$ edge solution corresponds to the situation in which $\hat{K}=1$. As a result, equations (3.12) reduce to

5.11 $$\frac{{\hat{t}}^{3}}{{\hat{Q}}_{0}^{2}}=\frac{\alpha^{5}{\hat{C}}^{10}}{B{\left(1,\hat{C},\alpha \right)}^{2}},$$

which determines the $K\tilde{K}$ edge solution. The solution depends on the parameter $\hat{t}/{\hat{Q}}^{2/3}$, which can be rewritten using (5.2) and (5.3) as *τϕ*^5/6^. In this case, the dimensionless time that quantifies the $K\tilde{K}$ transition is

5.12 $$\tau_{k\tilde{k}}=\frac{t}{t_{k\tilde{k}}}=\tau \phi^{5/6},\;t_{k\tilde{k}}={\left(\frac{K^{\prime 4}Q_{0}}{E^{\prime 4}C^{\prime 5}}\right)}^{2/3}.$$

The associated boundaries of the $K\tilde{K}$ transition, shown in figure 1, are calculated numerically as

5.13 $$\tau_{k\tilde{k}}^{\left(1\right)}\approx 5.96\times 10^{-8},\;\tau_{k\tilde{k}}^{\left(2\right)}\approx 4.81\times 10^{2},$$

where $\tau_{k\tilde{k}}^{\left(1\right)}$ corresponds to the beginning of the $K\tilde{K}$ transition (near the *K* vertex), whereas $\tau_{k\tilde{k}}^{\left(2\right)}$ corresponds to the end of the transition (near the $\tilde{K}$ vertex).

*Transition along the* $\tilde{M}\tilde{K}$ *edge.* To quantify the boundaries between the $\tilde{M}$ and $\tilde{K}$ vertices, it is necessary to consider the $\tilde{M}\tilde{K}$ edge transition. This $\tilde{M}\tilde{K}$ edge solution corresponds to the situation in which *η*=0 or $\hat{C}\gg 1$. Given that $g_{\delta }(0,\hat{C})=1/(\beta_{\tilde{m}}^{4}\hat{C})$ for large $\hat{C}$ (where $\beta_{\tilde{m}}=4/15^{1/4}/(\sqrt{2}-1{)}^{1/4}$), equations (3.12) and (3.14) can be reduced to

5.14 $$\frac{\hat{t}}{{\hat{Q}}_{0}^{2}}=\frac{\beta_{\tilde{m}}^{16}{\hat{K}}^{16}}{B{\left(2\alpha ,\frac{3}{2}\right)}^{2}},$$

which determines the $\tilde{M}\tilde{K}$ edge solution. The solution depends on the parameter $\hat{t}/{\hat{Q}}^{2}$, which can be rewritten using (5.2) and (5.3) as *τϕ*^−1/2^. In this case, the dimensionless time that quantifies the $\tilde{M}\tilde{K}$ transition is

5.15 $$\tau_{\tilde{m}\tilde{k}}=\frac{t}{t_{\tilde{m}\tilde{k}}}=\tau \phi^{-1/2},\;t_{\tilde{m}\tilde{k}}={\left(\frac{\mu^{\prime 2}E^{\prime 6}C^{\prime }Q_{0}}{K^{\prime 8}}\right)}^{2}.$$

The associated boundaries of the $\tilde{M}\tilde{K}$ transition, shown in figure 1, are calculated numerically as

5.16 $$\tau_{\tilde{m}\tilde{k}}^{\left(1\right)}\approx 4.18,\;\tau_{\tilde{m}\tilde{k}}^{\left(2\right)}\approx 2.01\times 10^{11},$$

where $\tau_{\tilde{m}\tilde{k}}^{\left(1\right)}$ corresponds to the beginning of the $\tilde{M}\tilde{K}$ transition (near the $\tilde{M}$ vertex), whereas $\tau_{\tilde{m}\tilde{k}}^{\left(2\right)}$ corresponds to the end of the transition (near the $\tilde{K}$ vertex).

## 6. Comparison with numerical solution

To check the accuracy of the developed approximate solution, it is necessary to compare predictions of the approximation to a reference solution. In this study, the reference solution is calculated numerically by solving the original equations governing the problem of a radial hydraulic fracture (2.2) and (2.3). One of the key features of the scheme is the ability to capture multiscale tip behaviour by using the tip asymptotic solution as a propagation condition. Brief description of the numerical scheme is given in appendix B.

Because §4 examined accuracy of the approximation at the limiting cases of vertex solutions, the aim of this section is to check the accuracy in the transition regions. For this reason, the choice of the parameters for comparison is *ϕ*={10^−18^,10^−15^,10^−12^,10^−9^,10^−6^,10^−3^,1, 10^3^,10^6^} and 10^−7^<*τ*<10^7^, which cover the transition region according to figure 1. It is important to note that the numerical solution is more difficult to obtain near the *K* and $\tilde{K}$ vertices, because the pressure is nearly constant, in which case the fluid flow within the fracture becomes irrelevant. At the same time, because the approximate width solution is exact at the *K* and $\tilde{K}$ vertices (and also $K\tilde{K}$ transition), the approximation is expected to be very accurate in these regions. In addition, the numerical scheme becomes less computationally efficient (i.e. requires finer mesh for convergence) near the $\tilde{M}$ vertex solution. This occurs, because the efficiency becomes very small, in which case most of the injected fluid is leaks into the formation. The accuracy of the approximation at the $\tilde{M}$ vertex, however, was evaluated earlier and there is no need to check it again with the numerical solution.

**Figure 2.**
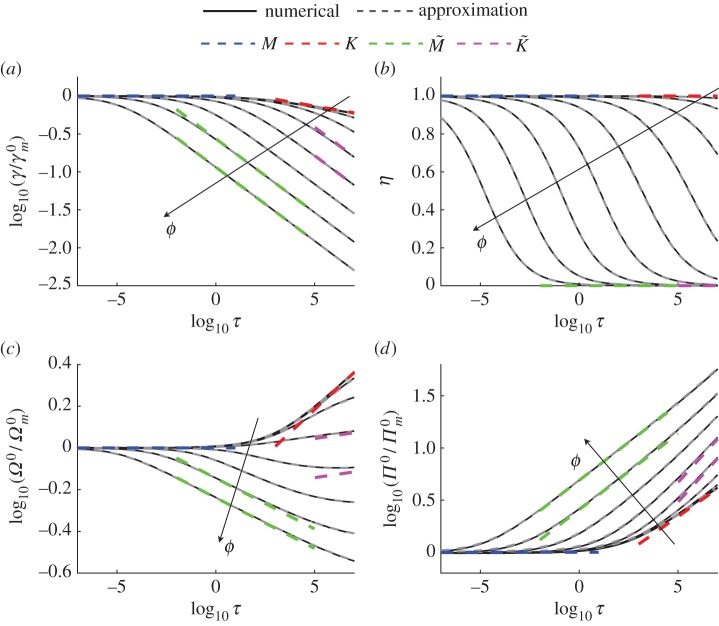
Comparison between the numerical solution (solid black lines) and the approximation (dashed grey lines) in terms of time histories of: (*a*) fracture radius, (*b*) efficiency, (*c*) width at the wellbore (*ρ*=0) and (*d*) fluid pressure at *ρ*=0.5. The fracture radius, width and pressure are normalized by the *M* vertex solution (6.1). Results are shown for *ϕ*={10^−18^,10^−15^,10^−12^,10^−9^,10^−6^,10^−3^,1,10^3^,10^6^}, where arrows indicate the directions in which the curves shift as the leak-off parameter *ϕ* increases. Dashed blue, red, green and magenta lines indicate the *M*, *K*, $\tilde{M}$ and $\tilde{K}$ limiting solutions, respectively.

Figure 2 shows comparison between the developed approximate solution (dashed grey lines) and the numerical solution (solid black lines) in terms of time histories of the fracture radius (panel (*a*)), efficiency (panel (*b*)), width at the wellbore (panel (*c*)) and pressure at *ρ*=0.5 (panel (*d*)), all for different values of *ϕ* (arrows indicate the direction in which the curves shift as *ϕ* increases). The fracture width and pressure are evaluated as

$$\Omega^{0}=\Omega (0,\tau ),\;\Pi^{0}=\Pi (0.5,\tau ),$$

where the pressure is not evaluated at the origin owing to a singularity located at that point. All the quantities are normalized by the *M* vertex solution (4.6), which can be written in terms of the scaling (5.1) and (5.3) as

6.1 $$\Omega_{m}^{0}(\tau )=\Omega_{m}(0)\tau^{1/9},\;\Pi_{m}^{0}=\Pi_{m}(0.5)\tau^{-1/3},\;\gamma_{m}^{0}=\gamma_{m}\tau^{4/9}.$$

The purpose of such normalization is to achieve better visual separation between the curves in figure 2. The vertex solutions are shown by the blue (*M* vertex), red (*K* vertex), green ($\tilde{M}$ vertex) and magenta ($\tilde{K}$ vertex) lines. All the solutions originate near the *M* vertex (i.e. the blue lines), and then transition to either *K*, $\tilde{K}$, $\tilde{M}$ or intermediate solution. Note that, eventually, all solutions (except the *ϕ*=0 case) reach the $\tilde{K}$ limiting case, as can be clearly seen from figure 1.

As can be seen from figure 2, there is no visual difference between the numerical solution and the approximation, which indicates that the developed approximate solution is accurate. Despite this fact, it is still important to quantify the level of accuracy of the approximation. To help doing this, the error is defined as

6.2 $$\text{Error}=\sqrt{\left\langle {\left[\frac{A_{\text{apr}}-A_{\text{num}}}{A_{\text{apr}}}\right]}^{2}\right\rangle },$$

where 〈⋅〉 indicates average over all time points, subscripts ‘apr’ and ‘num’ correspond to the approximation and numerical solution respectively, whereas *A* is either radius *γ*(*τ*), efficiency *η*(*τ*), wellbore width *Ω*^0^=*Ω*(0,*τ*) or pressure *Π*^0^=*Π*(0.5,*τ*).

**Figure 3.**
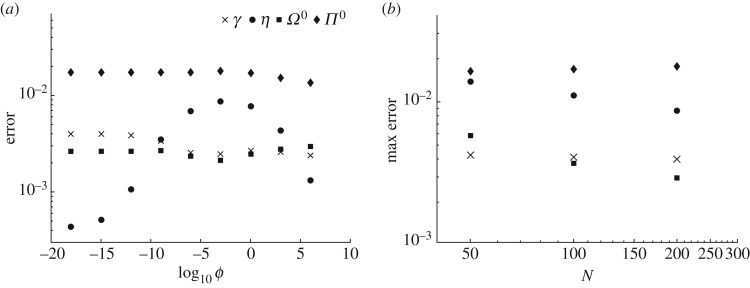
(*a*) Time-average discrepancy between the developed approximation and the numerical solution calculated using (6.2) for the fracture radius *γ*(*τ*), efficiency *η*(*τ*), wellbore width *Ω*^0^=*Ω*(0,*τ*) and pressure *Π*^0^=*Π*(0.5,*τ*). (*b*) Variation of the maximum error (maximum is calculated over *ϕ*) versus number of mesh points *N* of the numerical solution for the fracture length *γ*, efficiency *η*, wellbore width *Ω*^0^ and pressure *Π*^0^.

Figure 3*a* shows the error that is calculated using (6.2) for different values of the parameter *ϕ* for the finest mesh (for numerical solution) considered. The magnitude of the error for *γ*, *Ω*^0^ and *Π*^0^ does not vary notably versus *ϕ*, which indicates that the interpolation procedure for λ (4.29) is sufficiently accurate. The accuracy of all quantities, but the pressure, is under a per cent. The pressure is less accurate because it is calculated using the elasticity integral (2.3), which is very sensitive to the fracture width profile. To better understand the effect of mesh size that is used to calculate numerical solution, figure 3*b* shows the maximum error versus the number of spatial discretization points of the numerical solution *N*. Here the maximum is calculated for different values of *ϕ*, which can be obtained from figure 3*a* and its analogues for different meshes. Results in figure 3*b* demonstrate that error for the fluid pressure *Π*^0^ and fracture length *γ* reached the plateau values, but the efficiency *η* and the wellbore width *Ω*^0^ did not reach their respective plateau values for *N*=200. Despite this fact, it is still clear that the error of the developed approximation is under a per cent (for the considered error measure), which is sufficient for most practical cases.

**Figure 4.**
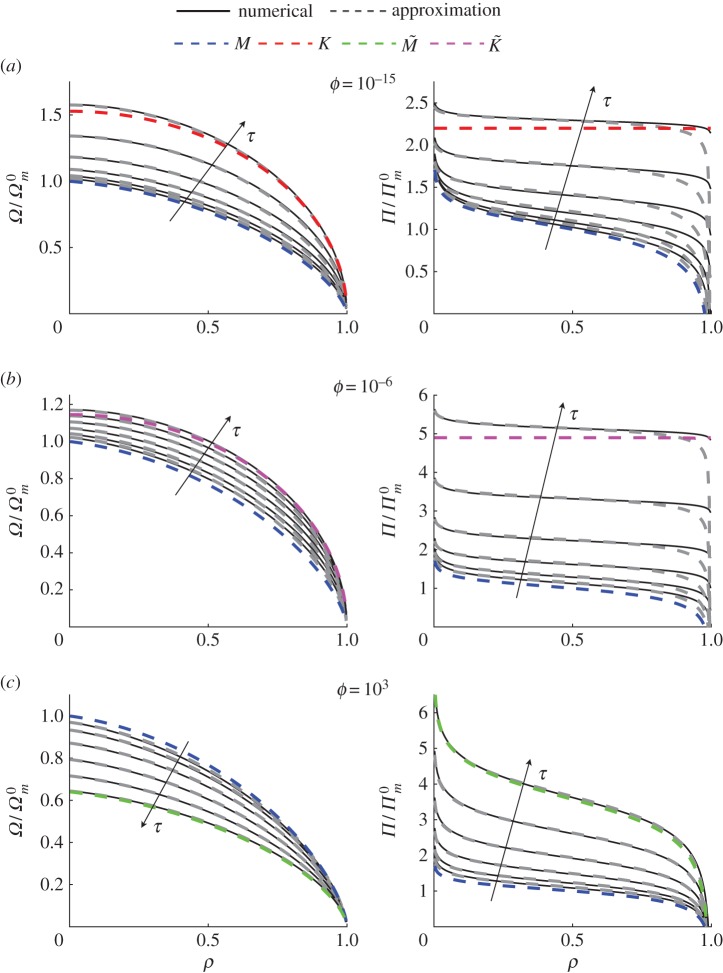
Fracture width and fluid pressure spatial variations calculated numerically (solid black lines) and obtained from the approximate solution (dashed grey lines) for: (*a*) *ϕ*=10^−15^ and *τ*={1,10,10^2^,10^3^,10^4^,10^5^}, (*b*) *ϕ*=10^−6^ and *τ*= {10,10^2^,10^3^,10^4^,10^5^,10^6^}, (*c*) *ϕ*=10^3^ and *τ*={10^−4^,10^−3^,10^−2^,10^−1^,1,10}. The fracture width and pressure are normalized by the *M* vertex solution (6.1). Dashed blue, red, green and magenta lines indicate the *M*, *K*, $\tilde{M}$ and $\tilde{K}$ limiting solutions, respectively.

In order to check accuracy of the pressure and width spatial variations, figure 4 plots pressure and width predictions stemming from the approximate solution (dashed grey lines) and those calculated numerically (solid black lines) for *ϕ*=10^−15^ (panel (*a*)), *ϕ*=10^−6^ (panel (*b*)) and *ϕ*=10^3^ (panel (*c*)). Both the pressure and the width are normalized by the *M* vertex solution (6.1) for convenience. The vertex solutions are shown by the blue (*M* vertex), red (*K* vertex), green ($\tilde{M}$ vertex) and magenta ($\tilde{K}$ vertex) lines for the first and the last value of *τ*. For the *ϕ*=10^−15^ case (panel (*a*)), the solution transitions from the *M* vertex and almost reaches the *K* vertex. For the *ϕ*=10^−6^ case (panel (*b*)), the solution transitions from the *M* vertex and approaches the $\tilde{K}$ vertex. For the *ϕ*=10^3^ case (panel (*c*)), the solution transitions from the *M* vertex to the $\tilde{M}$ vertex. These observations agree with the results shown in figure 1. Fracture width variations are consistently accurate, whereas the fluid pressure estimations deviate notably in the tip region. This is the weakest point of the approximate solution, and, therefore, it should not be used to evaluate pressure for ρ≳0.8. There might be a possibility to overcome this error by using (2.2) to estimate the pressure field. This, however, is a minor correction to the developed procedure and therefore is beyond the scope of this study.

## 7. Summary

This paper presents a closed-form approximate solution for a penny-shaped hydraulic fracture, whose behaviour is governed by a simultaneous interplay of fluid viscosity, fracture toughness and fluid leak-off into the formation. This development is made possible by combining the approximation for the fracture width profile, which uses the multiscale tip asymptotic solution in combination with the global fluid volume balance. First, the limiting regimes of the approximation are analyzed and the results are compared with the existing solutions. Then, a solution map is presented. This solution map indicates the areas of applicability of the limiting solutions and allows one to easily determine whether a problem with given parameters corresponds to one of the limiting cases or not. In addition, the developed solution captures the scaling that is associated with the transition from one limiting solution to another. In order to estimate accuracy of the approximate solution, a reference numerical solution is constructed. The latter numerical solution uses a moving mesh together with the implicit time stepping and a multiscale tip asymptotic solution to locate the moving fracture front. Results indicate that predictions of the fracture width and radius by the developed approximation are accurate to within a fraction of a per cent. The fluid pressure results are less accurate and lie within 2% error if measured half-way to the fracture tip. The importance of the developed approximate solution is twofold. First, it can be used to estimate fracture radius very quickly, in which case it can be used for a rapid fracture design calculations. Second, it can be used as a reference solution to test accuracy of more complex hydraulic fracture simulators (such as non-axisymmetric or non-planar), and can also be used as an initial condition for the aforementioned numerical codes.

## Data accessibility

Matlab code that produces the closed-form solution for a radial hydraulic fracture that is described in this paper is available from the Dryad Digital Repository at http://dx.doi.org/10.5061/dryad.gh469 [47].

## Appendix A. Functions $g_{\delta }(\hat{K},\hat{C})$ and $\Delta (\hat{K},\hat{C})$

This appendix provides expressions for the functions $g_{\delta }(\hat{K},\hat{C})$ and $\Delta (\hat{K},\hat{C})$ that are used in the paper to approximate the solution for a semi-infinite hydraulic fracture. Note that these functions were obtained in [34,14].

With reference to the scaling (3.7) and the fact that $\dot{R\;}\;=\alpha R/t$, the function *f* can be introduced as

A 1 $$\begin{array}{rl} \hat{s} & =\frac{1}{3C_{1}(\delta )}\left[1-{\hat{K}}^{3}-\frac{3}{2}\hat{C}\hat{b}(1-{\hat{K}}^{2})+3{\hat{C}}^{2}{\hat{b}}^{2}(1-\hat{K})-3{\hat{C}}^{3}{\hat{b}}^{3}\ln\left(\frac{\hat{C}\hat{b}+1}{\hat{C}\hat{b}+\hat{K}}\right)\right]\\ & \equiv f(\hat{K},\hat{C}\hat{b},C_{1}), \end{array}$$

where $\hat{b}=C_{2}(\delta )/C_{1}(\delta )$ and

A 2 $$C_{1}(\delta )=\frac{4(1-2\delta )}{\delta (1-\delta )}\tan(\pi \delta ),\;C_{2}(\delta )=\frac{16(1-3\delta )}{3\delta (2-3\delta )}\tan\left(\frac{3\pi }{2}\delta \right).$$

The zeroth-order approximation for the solution can be written as

A 3 $$\hat{s}=f\left(\hat{K},\frac{3\beta_{\tilde{m}}^{4}}{4\beta_{m}^{3}}\hat{C},\frac{\beta_{m}^{3}}{3}\right)\equiv g_{0}(\hat{K},\hat{C}),$$

where $\beta_{\tilde{m}}=4/\left(15^{1/4}(\sqrt{2}-1{)}^{1/4}\right)$ and *β*_*m*_=2^1/3^3^5/6^. As mentioned in [34], the solution varies spatially as $w_{a}(s)\propto s^{\bar{\delta }}$, where $\bar{\delta }=\frac{1}{2}(1+\delta )$ and the power *δ* is given by

A 4 $$\delta =\frac{\beta_{m}^{3}}{3}\left(1+\frac{3\beta_{\tilde{m}}^{4}}{4\beta_{m}^{3}}\hat{C}\right)g_{0}(\hat{K},\hat{C})\equiv \Delta (\hat{K},\hat{C}),$$

which defines the function $\Delta (\hat{K},\hat{C})$ and leads to the relation $\bar{\delta }=\frac{1}{2}(1+\Delta (\hat{K},\hat{C}))$. By substituting (A4) into (A1), the *δ*-corrected solution (3.7) can be written as

A 5 $$\hat{s}=f\left(\hat{K},\hat{C}\hat{b}(\Delta (\hat{K},\hat{C})),C_{1}(\Delta (\hat{K},\hat{C}))\right)\equiv g_{\delta }(\hat{K},\hat{C}),$$

which defines the function $g_{\delta }(\hat{K},\hat{C})$.

## Appendix B. Numerical scheme

To construct the numerical scheme, equation (2.2) is rewritten using *ρ*=*r*/*R*(*t*) and scaling (5.1)–(5.3) as

B 1 $$\frac{\partial \Omega }{\partial \tau }-\frac{\rho V}{\gamma }\frac{\partial \Omega }{\partial \rho }-\frac{1}{\gamma^{2}\rho }\frac{\partial }{\partial \rho }\left(\rho \Omega^{3}\frac{\partial \Pi }{\partial \rho }\right)+\frac{\phi^{1/4}}{\sqrt{\tau -\tau_{0}(\gamma \rho )}}=\frac{1}{\gamma^{2}}\delta (\rho ).$$

where $V=\dot{\gamma }$, whereas the elasticity equation (2.3) is reduced to

B 2 $$\Pi (\rho ,\tau )=-\frac{1}{2\pi \gamma }\int_{0}^{1}M(\rho ,s)\frac{\partial \Omega (s,\tau )}{\partial s}\;ds,$$

where the kernel is given in (2.4).

The spatial coordinate *ρ* is discretized as $\rho_{j}=(\frac{1}{2}+j)\Delta \rho$, *j*=1…*N*, in which case $\rho_{1}=\frac{1}{2}\Delta \rho$ and $\rho_{N}=1-\frac{1}{2}\Delta \rho$, and the temporal coordinate *τ* is discretized uniformly on a logarithmic scale. Piecewise constant approximation for *Ω* is used, in which case $\Omega_{j}^{i}=\Omega (\rho_{j},\tau_{i})$ and the vector ***Ω***^*i*^ represents an array of values of $\Omega_{j}^{i}$ for all *j*. In this situation, the elasticity equation (B2) is discretized as

B 3 $$\Pi^{i}=C\Omega^{i},\;C_{mn}=\frac{1}{2\pi \gamma^{i}}\left[M(\rho_{m},\rho_{n}+\frac{1}{2}\Delta \rho )-M(\rho_{m},\rho_{n}-\frac{1}{2}\Delta \rho )\right].$$

Fluid balance (B1), on the other hand, is discretized using backward time difference as

B 4 $$\frac{\Omega^{i}-\Omega^{i-1}}{\Delta \tau }=B\Omega^{i}+A(\Omega^{i})\Pi^{i}+S^{i},$$

where term capturing the moving mesh is discretized as

$$\begin{array}{rl} {\left[B\Omega^{i}\right]}_{j} & =\frac{\rho_{j}V}{\gamma }\frac{\Omega_{j+1}^{i}-\Omega_{j-1}^{i}}{2\Delta \rho },\;j=2\ldots N-1,\\ {\left[B\Omega^{i}\right]}_{1} & =\frac{\rho_{1}V}{\gamma }\frac{\Omega_{2}^{i}-\Omega_{1}^{i}}{2\Delta \rho },\;{\left[B\Omega^{i}\right]}_{N}=-\frac{\rho_{N}V}{\gamma }\frac{\Omega_{N}^{i}+\Omega_{N-1}^{i}}{2\Delta \rho }, \end{array}$$

the lubrication term is discretized as

$$\begin{array}{rl} {\left[A(\Omega^{i})\Pi^{i}\right]}_{j} & =\frac{1}{\gamma^{2}\rho_{j}\Delta \rho }\left[\rho_{j+1/2}{\left(\Omega_{j+1/2}^{i}\right)}^{3}\frac{\Pi_{j+1}^{i}-\Pi_{j}^{i}}{\Delta \rho }-\rho_{j-1/2}{\left(\Omega_{j-1/2}^{i}\right)}^{3}\frac{\Pi_{j}^{i}-\Pi_{j-1}^{i}}{\Delta \rho }\right],\;j=2\ldots N-1,\\ {\left[A(\Omega^{i})\Pi^{i}\right]}_{1} & =\frac{\rho_{3/2}{\left(\Omega_{3/2}^{i}\right)}^{3}}{\gamma^{2}\rho_{1}\Delta \rho }\frac{\Pi_{2}^{i}-\Pi_{1}^{i}}{\Delta \rho },\;[A(\Omega^{i})\Pi_{N}^{i}=-\frac{\rho_{N-1/2}{\left(\Omega_{N-1/2}^{i}\right)}^{3}}{\gamma^{2}\rho_{N}\Delta \rho }\frac{\Pi_{N}^{i}-\Pi_{N-1}^{i}}{\Delta \rho }, \end{array}$$

where quantities at the mid points *j*±1/2 are calculated as an average between the corresponding values of these quantities, and the source/leak-off term is

$$S_{j}^{i}=-\frac{2\phi^{1/4}}{\Delta \tau }\left[\sqrt{\tau_{i}+\Delta \tau -\tau_{0}(\gamma \rho_{j})}-\sqrt{\tau_{i}-\tau_{0}(\gamma \rho_{j})}\right]+\frac{\delta_{1j}}{\pi \gamma^{2}\Delta \rho^{2}},\;j=1\ldots N-1,$$

where *δ*_1*j*_ is the Kronecker delta. The values of [***B******Ω***^*i*^]_*N*_ and [***A***(***Ω***^*i*^)***Π***^*i*^]_*N*_ are obtained by integration of (B1) over the last element and using the no-flux condition at the tip.

In order to capture multiscale behaviour near the fracture tip, the following propagation condition is used

$$\Omega_{N-1}^{i}=\Omega_{a}(\frac{3}{2}\gamma \Delta \rho ),$$

where *Ω*_a_ is the scaled tip asymptotic solution. The latter equation implies that the numerical solution follows the asymptotic solution from the penultimate element to the tip, and allows one to determine the propagation velocity *V* . To successfully use this condition, pressure at the tip element $\Pi_{N}^{i}$ is treated as an unknown. Because the tip asymptotic solution satisfies (3.7), then

B 5 $$\hat{s}=g_{\delta }(\hat{K},\hat{C}),\;\hat{K}=\frac{d^{1/2}}{\Omega_{N-1}^{i}},\;\hat{C}=\frac{2d^{3/2}\phi^{1/4}}{{\left(\Omega_{N-1}^{i}\right)}^{5/2}{\hat{s}}^{1/2}},\;V=\frac{\hat{s}{\left(\Omega_{N-1}^{i}\right)}^{3}}{d^{2}},$$

where $d=\frac{3}{2}\gamma \Delta \rho$ signifies the distance from the centre of the penultimate element to the tip. Equation (B 5) is solved for $\hat{s}$ using Newton’s method, and the velocity of propagation is calculated. Because the solution in the tip element follows the asymptotic solution, then it is possible to determine its average opening as

B 6 $$\Omega_{N}^{i}={\left(\frac{2}{3}\right)}^{(3+\delta )/2}\frac{2\Omega_{N-1}^{i}}{3+\delta }\frac{d}{\Delta \rho },$$

where it is used that *Ω*_*s*_∝*d*^(1+*δ*)/2^ and $\delta =\Delta (\hat{K},\hat{C})$. The factor 2/3 ensures that the calculation is performed only within the tip element (and does not continue the middle of the penultimate element). Leak-off in the tip element is calculated by taking *τ*−*τ*_0_=*γ*(1−*ρ*)/*V* , in which case

B 7 $$S_{N}^{i}=-2\phi^{1/4}\sqrt{\frac{V}{\gamma \Delta \rho }}.$$

The numerical scheme consists of solving (B3)–(B7) for $\Omega_{j}^{i}$ (for *j*=1…*N*−1) and $\Pi_{N}^{i}$, which is done iteratively for each time step. The fracture radius is then updated as *γ*^*i*^=*γ*^*i*−1^+*V* *Δτ*.
